# Stressor-Dependant Changes in Immune Parameters in the Terrestrial Isopod Crustacean, *Porcellio scaber*: A Focus on Nanomaterials

**DOI:** 10.3390/nano11040934

**Published:** 2021-04-06

**Authors:** Craig Mayall, Andraz Dolar, Anita Jemec Kokalj, Sara Novak, Jaka Razinger, Francesco Barbero, Victor Puntes, Damjana Drobne

**Affiliations:** 1 Biotechnical Faculty, University of Ljubljana, 1000 Ljubljana, Slovenia; craig_mayall@hotmail.co.uk (C.M.); Andraz.Dolar@bf.uni-lj.si (A.D.); anita.jemec@bf.uni-lj.si (A.J.K.); Sara.Novak@bf.uni-lj.si (S.N.); 2 Plant Protection Department, Agricultural Institute of Slovenia, Ljubljana, Slovenia; jaka.razinger@kis.si; 3 Institut Català de Nanociència i Nanotecnologia (ICN2), CSIC and The Barcelona Institute of Science and Technology (BIST), Campus UAB, 08193 Bellaterra, Barcelona, Spain; fra.barbero@gmail.com (F.B.); victor.puntes@icn2.cat (V.P.)

**Keywords:** gold nanoparticles, cerium nanoparticles, woodlice, immune response, haemocyte

## Abstract

We compared the changes of selected immune parameters of *Porcellio scaber* to different stressors. The animals were either fed for two weeks with Au nanoparticles (NPs), CeO_2_ NPs, or Au ions or body-injected with Au NPs, CeO_2_ NPs, or lipopolysaccharide endotoxin. Contrary to expectations, the feeding experiment showed that both NPs caused a significant increase in the total haemocyte count (THC). In contrast, the ion-positive control resulted in a significantly decreased THC. Additionally, changes in phenoloxidase (PO)-like activity, haemocyte viability, and nitric oxide (NO) levels seemed to depend on the stressor. Injection experiments also showed stressor-dependant changes in measured parameters, such as CeO_2_ NPs and lipopolysaccharide endotoxin (LPS), caused more significant responses than Au NPs. These results show that feeding and injection of NPs caused an immune response and that the response differed significantly, depending on the exposure route. We did not expect the response to ingested NPs, due to the low exposure concentrations (100 μg/g dry weight food) and a firm gut epithelia, along with a lack of phagocytosis in the digestive system, which would theoretically prevent NPs from crossing the biological barrier. It remains a challenge for future research to reveal what the physiological and ecological significance is for the organism to sense and respond, via the immune system, to ingested foreign material.

## 1. Introduction

The role of the immune system is to distinguish between the self and non-self and to choose the most effective response to exogenous or endogenous threats, in order to maintain the integrity and homeostasis of the organism [1,2,3]. The immune recognition and response mechanisms of crustaceans depend entirely on the innate immune system.

The most prominent and well-studied cellular effector reactions of the innate immune system are phagocytosis, nodulation, encapsulation, cell-mediated cytotoxicity, and clotting. Cellular responses act in conjunction with humoral factors [4].

In crustaceans, in response to natural infections and challenges that mimic natural infections, such as lipopolysaccharide endotoxin (LPS), the response mechanisms and the recognition system that governs them are well -studied [5,6,7,8,9,10]. However, much less is known about the immune effector reactions in response to other challenges, such as pollutants [11]. Recently, the research on adverse or beneficial interactions between engineered nanomaterials and model organisms has provided a new model system to study if and how organisms sense and respond to novel engineered material of sizes comparable to those of viruses or bacteria [3].

Terrestrial isopods are a valuable model organism to study defences against challenges to homeostasis, as isopoda are the only order of crustacean able to occupy freshwater, marine, and terrestrial environments, which indicates that they have been exposed to a large variety of stressors during their evolution [12].

Isopods, as well as other arthropods, primarily rely on the cuticle as a sensing and defence system and the innate immune system to cope with different challenges to homeostasis. The cuticle is the relatively thin but tough and flexible layer of noncellular material that is capable of inducing many biochemical processes and able to respond to different environmental cues [13].

Haemocytes play a central role in the internal defence of the animal, although their role and function are not solely limited to purely immunogenic responses. In oysters, haemocytes were seen to participate in calcium carbonate shell crystal production, transportation, and shell regeneration [14,15]. Moreover, haemocytes in a range of organisms have been found to be involved in muscle fibre degeneration and regeneration; adult neurogenesis; and the digestion, storage, and distribution of nutrients [16,17,18,19]. The total haemocyte count (THC) is traditionally taken as a measure of an organism’s change in immunocompetence and is used in assessing the stress or health status in crustaceans [20,21,22,23]. It has been shown that the THC can vary not only in response to infection but also due to environmental stresses and endocrine activity during the moulting cycle [24,25].

An integral component of the innate immune system is the extracellular phenoloxidase (PO) cascade, which is part of humoral response that catalyses the formation of cytotoxic intermediates quinones, essential precursors for melanisation and sclerotisation [26,27,28,29]. Melanin accumulates at wound sites and around invading microorganisms [30]. It is not only involved in innate immune responses through melanotic encapsulation, it is also critical for cuticle tanning, which has been extensively studied in insects [19]. The PO cascade also has a role is ecdysis and exoskeleton formation [28].

The primary source of PO activity comes from the haemocytes [8,31]. However, prophenoloxidase (proPO) transcripts have been detected in nonhaemocyte cells too, including the hepatopancreas, stomach epithelium, anterior midgut caecum, glia cells in the nervous cord, and neurosecretory cells in ganglions [27,32]. Besides the plasma PO enzyme, literature also reports the existence of haemocyanin-derived PO (Hd–PO) activity [33,34]. Therefore, in isopods, PO-like activity in the haemolymph is usually shown, because, at this point, it is impossible to distinguish whether the observed activity is purely due to the plasma PO enzyme or haemocyanin-derived [5,33].

Activity of PO is taken as one of most frequently used parameters of assessing immune system activity. Phenoloxidase (PO) activity is increased as a result of clotting process’s, phagocytosis, encapsulation of foreign material, antimicrobial action, and cell agglutination [35]. The moult cycle, for example, is also a source of variation of phenoloxidase activity and correlates with the presence of large granular cells, although this appears to be species-specific [36,37]. Correlative measures of phenoloxidase (PO) activity and resistance to infection against vibriosis have been also investigated during the moult cycle [38]. In addition, it is documented in insects that, when soft cuticles are damaged, the pro-enzymes can become activated, [39] and the active enzymes can take part in both wound healing and in defending against invading microorganisms. The PO cascade not only results in the formation of cytotoxic pigment precursors, including quinones, quinone methides, and semiquinones, but also reactive oxygen species (ROS) and reactive nitric species (RNS), such as nitric oxide (NO) [40].

The concentration of NO is another well-studied and often used measure of immune system activity. The first evidence for NO to have a role as an humoral molecule in an invertebrate was provided by Radomski et al. (1991) [41]. Both RNS and ROS are produced by activated phagocytic cells. These molecules are involved in the breaking down of phagocytosed infectious agents such as viruses and bacteria, as well as dead and dying cells [42,43,44]. Otherwise, at lower concentrations, NO is involved in diverse housekeeping functions, such as neurotransmission, mucus secretion, and in controlling certain activities of the circulatory system, while at higher concentrations, NO is cytotoxic [45].

In the work presented here, we have used different types of immune challenges, i.e., lipopolysaccharide (LPS) and two types of nanoparticles and metal salt. LPS is a constituent of the cell membrane of Gram-negative bacteria. It has been widely used in investigating the immune function of a spectrum of model organisms [46,47,48,49]. For nanoparticles (NPs), two particles with well-known and frequently studied biological interactions were selected. Metal salt was used as a positive control to compare the effect of different forms of a metal (ions and nanoparticles).

Gold nanoparticles (Au NPs) have been used extensively in different experimental studies as their size, shape, and surface chemistry can be readily manipulated [50]. Due to their unique properties, they have a wide potential for applications in industry and biomedicine [50,51]. Numerous previous studies have found Au NPs to be relatively inert and nonharmful to organisms [52,53]. While some authors have suggested Au NPs have anti-inflammatory and antiangiogenic effects [54,55,56], others have shown Au NPs have toxic potential and can modulate immune processes in vitro and in vivo in some studies [50,54,57].

CeO_2_ NPs also represent nanoparticles with great industrial potential, due to their catalytic and redox properties [58,59]. Compared to Au NPs, they are more often considered as biologically potent and can induce oxidative stress; inflammation; DNA damage; and affect reproductive capability, growth, and the survival of exposed animals [59,60]. In the study of Kos et al. (2017), honeybees were exposed to the very same CeO_2_ NPs as in the present study. Significant alterations in Acetylcholinesterase and glutathione-S-transferase activities were evidenced at concentration 2 mg/L. However, in another experiment testing the toxicity of Au NPs and CeO_2_ NPs on earthworms, where the same exposure duration (as this paper) was used, CeO_2_ NPs showed a low toxic potential [59]. CeO_2_ NPs also possess dual properties, contrary to adverse effects some studies report on their antioxidant and anti-inflammatory properties [61,62].

Both CeO_2_ NPs and Au NPs have low dissolution rate [54,58]; therefore, the particles, and not the dissolved ions, are primarily responsible for the biological effect.

We provide a comparison of the responses of the innate immune system of terrestrial isopod *P. scaber* to different types of challenges to homeostasis (Au and Ce nanoparticles, salt, and LPS). We have analysed and compared some well-studied immune parameters (THC, haemocyte viability, PO-like activity, and NO concentration) after dietary exposure and in the case of body injection [63].

The aim of our work was to investigate if and how the most well-known and studied parameters of the immune system respond to nanomaterials in the food and to compare this response to that when nanomaterials are injected directly into the haemolymph of a terrestrial isopod, *Porcellio scaber*. Our focus was also directed towards comparing the response to nanoparticles vs. ions in the feeding experiment and towards comparing the response to nanoparticles vs. LPS in body injection experiments. We hypothesise that the response to NPs directly injected into the haemolymph or consumed with food is substantially different. We discuss the differences in response to the selected types of noninfectious immune system challenges in a model terrestrial invertebrate species.

## 2. Materials and Methods

### 2.1. Nanoparticle Synthesis and Characterisation

**Figure A1:**
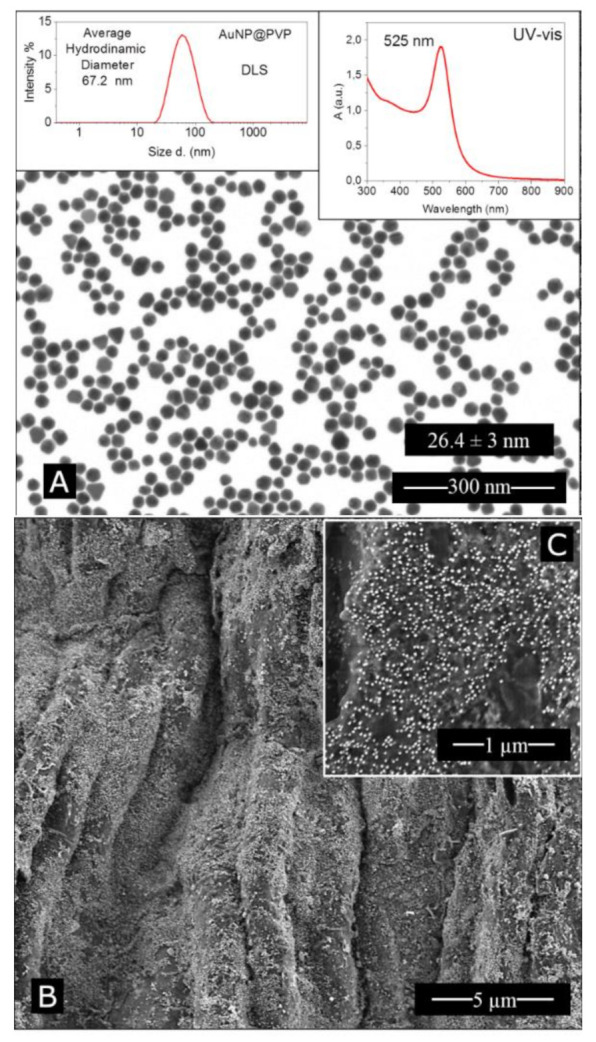
Polyvinylpyrrolidone coated Au NP physicochemical characterisation. (**A**) Bright field—scanning transmission electron microscopy (STEM); dynamic light scattering measurement of the Au NPs (Top left); UV–Vis spectra (Top right). (**B**,**C**) Scanning electron microscopy (SEM) of dried leaves covered with PVP Au NPs.

Au NPs were synthesised by the Catalan Institute of Nanoscience and Nanotechnology (ICN2). Au NPs (26.4 ± 3 nm) were synthesised according to the seeded-growth method developed and detailed in Bastús et al. (2011) [64]. Au NPs were purified and surface coated in PVP following the procedure outlined in Alijagic (2020) to produce PVP–Au NPs, referred to in this paper as Au NPs [65]. Dynamic light scattering (DLS) analysis showed the particles had an average hydrodynamic diameter of 67.2 nm, and laser doppler anemometry showed a Z potential of−3.9 ± 0.8 mV (pH 5.5, conductivity 0.9 mS/cm) (Malvern Zetasizer Nano ZS, Malvern Panalytical Ltd, Worcester, UK). Diameters were reported as distribution by intensity calculated by non-negative least squares (NNLS). A typical UV–Vis spectrum profile with a surface plasmon resonance band peaking at 525 nm showed the particles were monodispersed (Agilent Cary 60 spectrophotometer, Santa Clara, CA, USA). Tetrachloroauric (III) acid trihydrate (99.9% purity), sodium citrate tribasic dihydrate (≥99%), and polyvinylpyrrolidone (55 KDa) were purchased from Sigma–Aldrich. Particle characterisation can be found in Figure A1.

Stabiliser-free, uncoated spherical CeO_2_ NPs as an aqueous dispersion in dH_2_O (batch number PROM-CeO2-20 nm-2306/5a) were supplied by NanoMILE PROM (Promethean Particles, Nottingham, UK, http://www.prometheanparticles.co.uk/ 2 April 2021) within the framework of the EU FP7 NanoMILE project. The CeO_2_ NPs were synthesised using supercritical fluid synthesis, followed by a washing step postsynthesis to remove unreacted species. The mean particle diameter (TEM) was 4.7 ± 1.4 nm (JEOL JEM2100F, Tokyo, Japan), the Z-average size was 172.1 ± 1.705 nm, the polydispersity index (PDI) was 0.272 ± 0.009, and the zeta potential was 50.3 ± 0.719 mV (Malvern Zetasizer 5000, Malvern Panalytical Ltd, Worcester, UK). Further particle characterisation can be found in Kos et al. (2017) [66].

### 2.2. Experimental Animals

*Porcellio scaber* were collected from a compost heap in noncontaminated, pollution-free garden in Kamnik, Slovenia. Prior to the experiment, animals were cultured for a several months under constant temperature (20 ± 2 °C) and illumination (light:dark 16:8 h) regime in a climate-controlled chamber at the University of Ljubljana. Animals were kept in a glass terrarium filled up with a humus soil (moistened at 40% of the water holding capacity; WHC) and fed with dry leaves of common hazel (*Corylus avellana*), common alder (*Alnus glutinosa*), and carrots, as described by Jemec Kokalj et al. (2018) [67]. Adult animals of both sexes with body mass greater than 25 mg were selected for the experiment, while individuals with evident signs of moulting, gravid females, and those with symptoms of bacterial or viral infection were excluded [5]. We have followed the the ARRIVE (Animal Research: Reporting of *In Vivo* Experiments) guidelines (http://www.nc3rs.org.uk/page.asp?id=1357) for reporting experiments with using living animals. Experiments with terrestrial isopods do not need to be approved by the ethics committee.

### 2.3. Feeding Experiment

The feeding experiment was carried out for 14 days in petri dishes with a single animal at constant temperature (20 ± 2 °C), illumination (light:dark 16:8 h) regime, and moisture in a climate-controlled chamber, a semichronic exposure period. The animals were fed dried leaves covered with either cerium oxide nanoparticles (CeO_2_ NPs), gold nanoparticles (Au NPs), or ionic gold (AuCl_3_) as salt control or pure dried leaves, which served as controls. Nanoparticles and metal ions were spread on a leaf at a concentration of 100 μg/g of dry weight (d.w.) leaf. This concentration was chosen, as it is lower than the previously observed lowest effect concentration yet higher than the estimated soil compartment concentration, in order to provide an example of the model response [68,69,70,71]. Animals’ weights were taken before and after the experiment; additionally, dry leaf weights after nanoparticle application at the start and end of the experiment were taken. During the experiment, animal faeces were removed from the petri dishes every 48 h in order to measure defecation rate and to reduce coprophagy. At the end of the 14-day experiment, fresh haemolymph was collected from the animals, according to Dolar et al. (2020), to measure the selected immune parameters [5]. Details on the number of animals used can be found in Table A1.

### 2.4. Injection Experiment

For injection experiments, animals were injected with 0.5 μL of the lipopolysaccharide (LPS), CeO_2_ NPs, Au NPs or Milli-Q water as a trauma control, using a 25 μL Hamilton microsyringe (700 series, 33 gauge, blunt tip) and a repeating dispenser (PB 600-1, Hamilton, Bonaduz, Switzerland). Cerium and Au NPs were suspended in Milli-Q water at a concentration of 0.3 μg/μL. A nonlethal dose of LPS (from *E. coli* O111:B4, Sigma-Aldrich, St. Louis, MO, USA) was used as a positive control for reference immune response (50 μg/μL). Injected animals were left for 48 h in a petri dish with a dry leaf for food at a constant temperature (20 ± 2 °C) and illumination (light: dark 16:8 h) regime and moisture in a climate-controlled chamber. Afterwards, fresh haemolymph was collected from the animals to measure the selected immune parameters. Details on the number of animals used can be found in Table A2.

### 2.5. Haemolymph Collection

A sterile syringe needle was used to puncture the intersegmental membrane on the dorsal side of the animal between the 5th and 6th segment; then a drop of haemolymph was withdrawn using glass microcapillary pipette. Where not enough haemolymph could be collected from a single animal, a pooled sample was prepared for nitric oxide and phenoloxidase-like measurements.

### 2.6. Immune Parameters

To determine haemocyte viability and total haemocyte count (THC), 3–5 μL of freshly collected haemolymph was diluted in 100 mM PPB (pH 7) up to a volume 28 μL, and then 2 μL of nigrosin dye was added (final concentration of 80μg/mL), which stains dead cells, while viable haemocytes remain unstained. After that, ten microliters of haemocyte suspension was loaded on to each chamber of the Neubauer haemocytometer to assess haemocyte viability and THC under a phase contrast microscope (Axio Vert.A1, Zeiss; magnification: 40×, Oberkochen, Germany). In order to fulfil the requirement for a statistically significant count, at least 100 cells per square were counted (according to the instructions of the haemocytometer). Each haemolymph sample measurement was performed in duplicate.

Nitric oxide (NO) levels in the haemolymph were measured using Griess reagent after a modified protocol Faraldo et al. (2005) [72]. Twenty microliters of a pooled haemolymph sample, collected from an average of 4 animals, was placed into 28 μL of 1% sulphanilamide dissolved in 5% phosphoric acid (H_3_PO_4_) and kept on ice. After that, 20 μL of this solution was diluted at a ratio of 1:1 (**v:v**) with 1% naphthylethylenediamine dihydrochloride (NEED, Sigma). The absorbance of NO was measured in a 384-well assay plate at 543 nm after a 5-min incubation period using a Cytation 3 imaging reader (Biotek, Winooski, VT, USA). Each haemolymph sample measurement was done in duplicate. NO concentrations (μM) were calculated from a standard curve for NaNO_2_ (2.5–200 μM).

Phenoloxidase (PO)-like activity was assessed photometrically in the haemolymph obtained from 1–3 animals, after a modified protocol described by Jaenicke et al. (2009) [34]. Five microliters of haemolymph was placed into 200 μL of Dulbecco’s phosphate buffered saline buffer (DPBS, pH 7.1–7.5), containing 4 mM dopamine hydrochloride (Sigma-Aldrich, St. Louis, MO, USA) and 2 mM sodium dodecyl sulphate (SDS, Sigma), necessary for in vitro PO activation. Forty microliters of this reaction mixture was pipetted into 384-well plate (Greiner Bio-One, Kremsmünster, Austria). The formation of a reddish–brown pigment, which is nonenzymatically synthesised from the dopamine catalytic product dopaquinon, was measured using a Cytation 3 imaging reader (Biotek, Winooski, VT, USA) at 475 nm and 25 °C for at least three hours. Haemolymph PO-like activity was calculated as the change in absorbance from the linear part of the absorbance slope per minute per haemolymph volume, normalised to control and expressed as a percentage [73]. PO-like activity assessment was done in duplicate for each haemolymph sample.

### 2.7. Statistical Analysis

Statistical analysis of the data was performed using OriginPro v2020 software program OriginLab, Northampton, MA, USA). The data was tested using Kruskal–Wallis test, followed by pairwise comparison of treatments with control group, using Mann–Whitney U-test. Values of *p* < 0.001 (***), *p* < 0.01 (**) and *p* < 0.05 (*) were considered as significantly different.

## 3. Results

### 3.1. Feeding Activity of Isopods and Mortality

**Figure 1:**
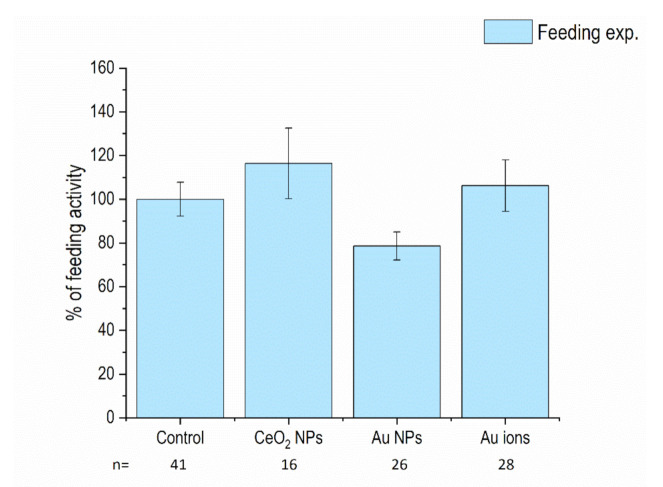
Feeding activity of isopods in comparison to control (%) after 14 days of feeding on CeO_2_ nanoparticle (NP)-, Au NP-, and Au ions-spiked leaves. Columns represent the mean (±SE). n = number of biological repeats in each column.

The feeding activity of isopods, calculated as the mass of leaves ingested per each animal’s mass, in two weeks, was not significantly altered in animals that were fed CeO_2_ NP- or Au NP-spiked leaves. In addition, Au ions (AuCl_3_) as a salt control for Au NPs did not induce any changes in feeding activity. No signs of animal mortality were evidenced (data not shown) (Figure 1). These data show that the tested concentration (100 μg/g d.w. leaf) did not induce significant adverse effects and can be considered not toxic.

### 3.2. NO Levels and PO-Like Activity

**Figure 2:**
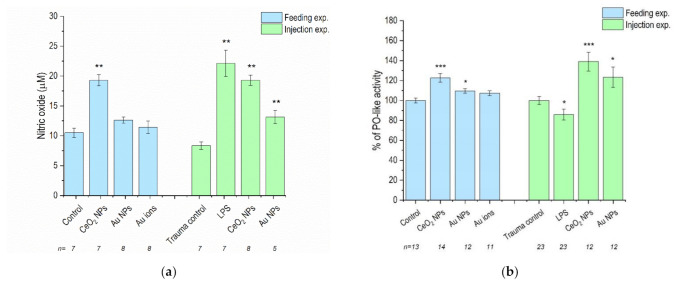
(**a**) Nitric oxide concentrations and (**b**) phenoloxidase (PO)-like activity in *P. scaber* exposed to CeO_2_ NP- and Au NP-spiked leaves (Feeding exp.) and injected with CeO_2_ NPs and Au NPs dispersion (Injection exp.). Au ions correspond to AuCl_3_, trauma control are animals injected with dH_2_O and lipopolysaccharide endotoxin (LPS); animals injected with 50 μg/μL LPS. Columns represent the mean (±SE). n represents the number of analysed samples. For nitric oxide (NO) measurements, on average, 4–5 animals were grouped into one sample, and for the PO-like measurements, 1–3 animals were grouped into 1 sample. (*) *p* < 0.05, (**) *p* < 0.01, (***) *p* < 0.001 in comparison to control (Kruskal–Wallis test followed by Mann–Whitney U-test).

Nitric oxide (NO) levels were significantly increased after the animals were fed or injected with CeO_2_ NPs, while Au NPs caused increases in NO levels only when injected. The observed increase was more significant in the case of CeO_2_ NPs, in comparison to Au NPs (Figure 2a).

PO-like activity was significantly increased in the case of both CeO_2_ NPs and Au NPs after feeding and injection. The increase was again more significant in response to CeO_2_ NPs (Figure 2b). The injection procedure, itself, had no effect on NO levels or PO-like activity, as evidenced by no significant changes in the trauma controls. Injection with LPS, a positive control, significantly increased the NO levels and caused a decrease in PO-like activity.

### 3.3. Haemocyte Count

**Figure 3:**
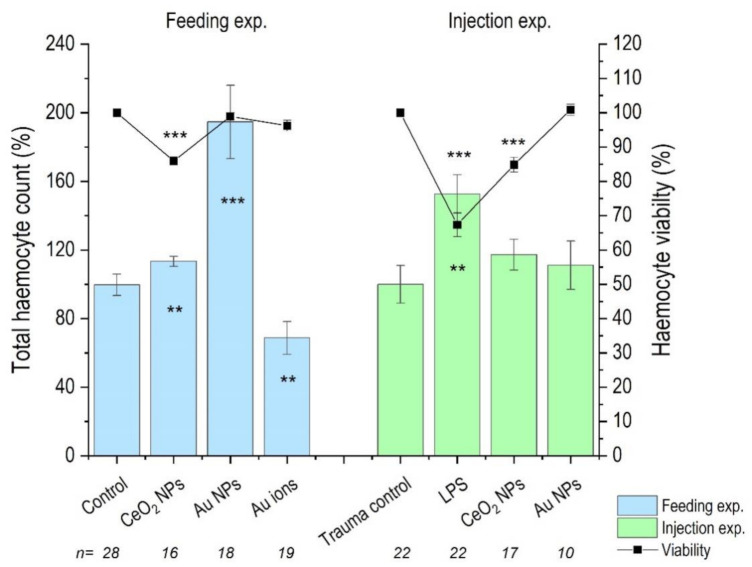
Total haemocyte count (THC) and haemocyte viability in *P. scaber* exposed to CeO_2_ NP- and Au NP-spiked leaves (Feeding exp.) and injected with CeO_2_ NPs and Au NPs dispersion (Injection exp.). Au ions correspond to AuCl_3_, trauma control are animals injected with dH_2_O and LPS; animals injected with 50 μg/μL. Columns represents mean (±SE). n represents the number of biological repeats. (**) *p* ≤ 0.01, (***) *p* ≤ 0.001 in comparison to control (Kruskal–Wallis test followed by Mann–Whitney U-test). When * is drawn inside the column, it refers to THC values.

The total haemocyte count (THC) was significantly increased in the case of CeO_2_ NPs and Au NPs, in comparison to control after feeding, but decreased in the case of Au ions. However, after injections, neither CeO_2_ NPs nor Au NPs affected the THC. The viability of the haemocytes was decreased after both feeding and injection CeO_2_ NP exposures, but Au treatments had no effect on viability after either exposure route. Injection, itself (evidenced as trauma control), had no effect on the THC or viability of haemocytes; however, both THC and viability were affected after LPS injection, serving as a positive control (Figure 3).

## 4. Discussion

We report a comparison of the responses of well-studied immune parameters (THC, haemocyte viability, PO-like enzyme activity, and NO concentration) of the terrestrial isopod *P. scaber* to different types of stressors (Au and CeO_2_ nanoparticles, salt, and LPS) after feeding exposure and after direct injection into the animal’s body. While injection is not a realistic exposure route for NPs, it can help to study consequences of direct biological interactions between NPs and isopod haemocytes inside the body (in vivo).

### 4.1. Feeding on Au NP-Dosed Diet

In animals fed for 14 days on a Au NP-dosed diet, we detected a significant increase in the THC, no effect on haemocyte viability, a slight increase in PO-like activity, and no change in the NO concentration. Literature explains such a response as immunostimultion by oral administration [74] or an indication of increased immune capacity [75]. Setyawan et al. (2018) also provided evidence that diet composition (crude fucoidan from three tropical brown algae i.e., Sargassum, Padina, and Turbinaria) was able to provoke increased PO, THC, and relative superoxide dismutase (SOD) activity in white shrimp [74]. In addition, in lobster, haemocyte levels, as well as the haemolymph protein levels, were found to be influenced by diet more than by temperature [76]. Pascual et al. (2004) reported that the amounts of dietary protein were positively corelated to the TCH response in *L. vannamei* juveniles [77]. In their study, shrimp fed an optimal level of protein had an elevated concentration of haemocytes, indicating that optimal dietary protein levels promoted blood cell synthesis [77]. In contrast, shrimp fed suboptimal protein had more proPO per cell, showing that shrimps could be enhancing the content of the haemocytes (i.e., proPO) as a response of cell deficit induced by imbalanced dietary protein [77]. There are also reports in different insect species that the THC increased when they were withheld food [78,79]. Furthermore, Matozzo et al. (2011) observed a positive relationship between the THC and PO activity in starved crabs. They hypothesised that the number of circulating haemocytes increased in starved crabs to allow them to mobilise energy reserves during starvation [80]. In addition, Sequeira et al. (1996) suggested that increases in the THC in Crustacea may be either due to a more active mobilisation of haemocytes from tissues into the haemolymph or a faster division of circulating haemocytes [81]. In line with our findings are reports of Muralisankar et al. (2014), who found an elevated THC and haemocyte production after ZnNP exposure to the freshwater prawn, *Macrobrachium rosenbergii*, within a certain exposure dose range [82]. We can conclude that increases of the THC or in PO-like activity are the result of a complex response of the organism responding to ingested material that is different from its usual diet but one that is not necessarily an indication of adverse effect. What this complex response is and why it has physiological or ecological consequences needs to be discovered.

There are only a few studies on the mechanism of how components in the diet challenge immune processes. In crustaceans, for example, the hepatopancreas, which comes into direct contact with ingested material (not protected by cuticle or peritrophic membrane), produces many proteins that contribute to humoral immunity, including the two most abundant proteins in the haemolymph, clotting protein and haemocyanin [83,84]. Both of these proteins play roles in the immune response [83,84], especially haemocyanin, which is believed to carry out phenoloxidase-like functions in *P. scaber* [34].

We suggest that ingested Au NPs could come into contact with hepatopancreatic cells, and the NPs’ environmental corona could be recognised by hepatopancreatic cells [85]. Since a feeding rate reduction was not detected in this study, food quality, and not food deprivation, must be the explanation for the increased THC or PO activity. As ingested Au NPs did not cause an increase in the NO concentration, we conclude that there is a lack of evidence of phagocytic activity of damaged material (damaged-self) [86]. In addition, with no changes in haemocyte viability, we speculate that the changes in immune parameters via hepatopancreas–humoral immunity communication/signalling are primarily not damage-related (damaged-self-related).

### 4.2. Feeding on CeO_2_ NP-Dosed Diet

In animals fed for 14 days on the CeO_2_ NP-dosed diet, increased PO-like activity and reduced viability of haemocytes was accompanied by a significant increase of NO activity, suggesting phagocytic activity of damaged or non-self material. However, NO is also produced in the PO cascade, which results in the formation of cytotoxic pigment precursors (quinones, quinone methides, and semiquinones) as well as reactive oxygen intermediates (ROI) and reactive nitrogen intermediates (RNI) [40].

In CeO_2_ NP-fed animals, we explain the increased THC, reduced haemocyte viability, increased PO-like activity, and increased NO concentration as an immune response, indicating either increased melanisation via increased PO-like activity and/or increased production of toxic radicals (via NO), which could occur either during haemocyte phagocytic activity or melanisation. This response to CeO_2_ NPs could be provoked due to cuticle (external surfaces) damage and subsequent melanisation of the cuticle either on the body surface or in the digestive system [13]. It is assumed that hepatopancreatic cells, as a possible contact site between cells and NPs, have probably not been damaged. If damage had occurred here, it would likely have been reflected in a reduced feeding rate, which was not seen in this study.

We speculate that the organism responded to microdamages or changes in the cuticle structure, which surrounds the gut epithelium, when exposed to CeO_2_ NP-dosed food. As reported by Parle et al. (2017), for insects, an injury that penetrates the epidermis results in a scab which is never resorbed [87].

In the case of the CeO_2_ NP-fed animals in our study, the increased THC and PO-like activity was accompanied by a significant reduction in the viability of haemocytes and a significant elevation in NO levels, suggesting phagocytosis [88], which may indicate a (micro)damage-related response. The response pattern of *P. scaber* to CeO_2_ NPs is significantly different from that in Au NP-fed animals, indicating a different type of interaction between the two particles and *P. scaber*. The Au NPs appeared more biologically inert than CeO_2_ NPs.

Even if CeO_2_ NPs demonstrated some biological reactivity, according to Giese et al. (2018), environmentally relevant concentrations of CeO_2_ NPs are those 100-fold lower that the one recorded to provoke toxicity—concentrations ranging from 100 to 3000 mg/kg or mg/L [89].

### 4.3. Feeding on Au Ions-Dosed Diet

As expected, the Au ions provoked a different type of response, as measured via the selected immune parameters, than the Au NP consumption. We detected a significant decline in the THC but no change in feeding, mortality, haemocyte viability, PO-like activity, or NO concentration. It is interesting that feeding on Au NPs provoked a completely opposite response compared to Au ions. Au NP particles in the diet caused significant increases in the THC and of PO-like activity, while Au ions in the diet resulted in a THC decline.

Similarly, a decrease in the number of circulating haemocytes (TCH) was demonstrated in mercury-exposed prawns [90] and in crabs after bacterial infection [91] or after temperature stress [92]. A decrease in the number of circulating haemocytes is explained as a consequence of haemocyte immobilisation in different tissues, as demonstrated in mercury-exposed prawns [90] and in crabs after bacterial infection [92,93]. Consequently, this leads to a stress-induced decrease in immunocompetence [94] and renders organisms more susceptible to disease. Le Moullac et al. (2000) reported that environmental stress from pollutants induced immunosuppression in crustaceans, in terms of haemocyte number, phagocytosis, and a change in PO activity [21]. Further, Quin et al. (2012) reported that the total haemocyte count in Cd-exposed groups was decreased significantly, when compared with the control groups of freshwater crab, *Sinopotamon henanense* [22]. In addition, Singaram et al. (2013) reported that 14 days of exposure to Hg at environmentally relevant concentrations suppressed THC, superoxide generation, phagocytosis, and PO activity, among other immune parameters [95].

Matozzo et al. (2011) hypothesised that increased PO activity in haemolymph from temperature-stressed crabs was a physiological response of animals to compensate for the lower THC, in order to increase immunosurveillance in both haemolymph and peripheral tissues [92], corroborating findings by Hauton et al. (1997) [96]. In our study, the PO-like activity was not changed to potentially compensate for the decreased THC. However, we confirm negative consequences of Au ion exposure on the THC, which is in line with findings after mercury exposure.

### 4.4. Injection of Stimuli into Dlood

The second approach in our study to challenge the immune parameters of isopods was by the injection of different stimuli (LPS or NPs) directly into the haemocoel and to leave the animals for 48 h to develop a response. A lipopolysaccharide (LPS) is an endotoxin, which is an integral component of the outer membrane of Gram-negative bacteria and, thus, can be used to mimic a bacterial infection [97].

In our study, injected LPS resulted in an increased THC, decreased haemocyte viability, the generation of NO, and reduced PO-like activity. Other authors have also reported alterations in the THC after LPS injection. In insects, adult male *Acheta domesticus* crickets, an LPS injection caused a significant decrease in the number of circulating haemocytes 2 h post injection, which was followed by an increase in haemocyte numbers 1 day after injection and then maintained levels for at least 7 days [73]. Similarly, Bogolio et al. (2000) reported that, after an initial decrease in haemocyte counts in juvenile prawns, a sublethal infection with *V. alginolyticus* induced higher haemocyte counts than controls at 8 days post-infection [98]. In an experiment by Lorenzon et al. (1999), multiple shellfish were injected with an LD50 dose of LPS *E. coli* [99]. In most of the species, the LPS caused an initial decrease in haemocytes (40% at 3–5 hpi), then an increase in haemocytes to control animal levels after 48 hpi (*Palaemon elegans*, *Crangon crangon*, and *Squilla mantis*). A challenge with LPS injection is also reported by Chiaramonte et al. (2019) on the sea urchin *Paracentrotus lividus* [100]. Here, LPS treatment significantly increased the number of coelomocytes after the first hour of treatment. At 6 and 24 h post-LPS treatment, values obtained did not significantly differ from the untreated controls. LPS injection in crayfish [48] caused an increase in haemocytes 2 h post-LPS injection, as well as a significant decrease in haemocyte viability. Furthermore, Xu et al. (2015), who exposed crab haemocytes to LPS, reported that LPS causes a decrease in haemocyte viability, due to the increase apoptosis rate [101]. They found that LPS damaged DNA and caused morphological changes in the haemocytes, resulting in cell shrinkage, nucleus membrane and chromatin fracturing, and the formation of apoptotic bodies [101].

Similar to our work is a study conducted with juvenile *Litopenaeus stylirostris*, which evaluated the impact of the injection of (1) formalin-killed *Vibrio penueicida* cells (vaccine) and (2) a sublethal infection with a moderately pathogenic strain of *Vibrio alginolyticus* on total haemocyte counts. After an initial decrease in haemocyte counts, both the vaccination and the sublethal infection induced higher haemocyte counts than controls. In order to investigate the possibility of increasing the number of circulating haemocytes in pond-reared juvenile shrimp, the vaccine was added to the diet. No effect on the haemocyte counts was observed over a 20-day feeding period [98].

Contrary to our expectations, only LPS injection caused changes in the TCH. However, the level of NO was increased in all three injection treatments: LPS, CeO_2_ NPs, and Au NPs. Literature reports that LPS stimulates non-self-induced nitric oxide generation involving nitric oxide synthase in haemocytes and the production of nitric oxide during phagocytosis as a cellular immune reaction to fight the pathogen [6]. Yeh et al. (2006) also postulate that the observed increase in nitric oxide synthase (NOS) and NO generation increases bacterial adhesion to haemocytes, and as such, LPS increases both haemocyte adhesion and the phagocytic activity of the haemocytes [102]. Gopalakrishnan et al. (2011) found that, in the crab *Scylla paramamosain,* LPS stimulation caused the levels of NO to increase from 3 h to 96 h post-injection [6]. In crayfish injected with LPS, after 1 h, a two-fold increase in NO levels was found, compared to animals injected with only saline [102]. Similarly, NP injection may also provoke phagocytic activity of the haemocytes, but only the CeO_2_ NPs affected their viability.

We also evidenced alterations of PO-like activity in all cases of injected stimuli. LPS injection caused a reduction of PO-like activity, while both injected NPs increased the PO-like activity. Our results are in line with those reported by Charles & Killian (2015), who found that, in crickets, LPS caused an increased THC and decreased PO activity at 7 days [73]. However, these results were with LPS from *S. marcensces* and not *E. coli* [73]. Contrary to our study, Gopalakrishnan et al. (2011) found that, in the crab *Scylla paramamosain,* LPS injection stimulation caused an increase in PO activity from 3 h to 48 h, while at the same time causing a decrease in the THC [6]. Salawu et al. (2016) suggested that the role of activated PO in the crab *Uca tangeri* was to limit the survival of Gram-negative bacteria upon entry into the haemolymph, and the PO is immediately necessary upon the LPS being identified, with there being an increase in activated PO in the haemolymph 10 min after exposure to LPS [103]. In our study, LPS caused increases in the THC but no increase in PO-like activity; in fact, PO-like activity was decreased again, showing a complex response to a stimulus, as evidenced also in the ingestion exposure.

To sum up, our results show that haemocyte viability and NO response is more severe in case of LPS and least in case of Au NPs (LPS > CeO_2_ NPs > Au NPs). Excessive NO production is explained by increased phagocytosis activity of cells [88]. Injection of CeO_2_ NPs and Au NPs resulted in the significant increase in PO-like activity and NO concentration. CeO_2_ NPs also caused a significant decrease in haemocyte viability, a response also seen after LPS injection, suggesting CeO_2_ NPs were causing more damage than the Au NPs to the haemocytes, with the former causing a higher instance of haemocyte death. The same, CeO_2_ NPs being more biologically potent than Au NPs, was observed in feeding exposure.

## 5. Conclusions

There are significant differences in the immune response after feeding or injection exposure to different stressors. In both types of exposure, feeding or injection of both NPs, increased the PO-like activity. However, injected LPS caused a reduction of PO-like activity. Increased PO-like activity is interpreted to lead to increased melanisation, which is needed for wound healing [29] but may have other functions, as well. Furthermore, NO was increased in all cases of injection, but only in the case of CeO_2_ NPs in the feeding experiment and was most significantly increased after LPS injection. NO is associated with the phagocytosis of non-self and damaged-self [86,104]. Therefore, we conclude that CeO_2_ NPs, and not Au NPs, were causing (micro)damage/alterations to the isopods’ tissues and cells, resulting in the increase in phagocytosis, as evidenced by NO levels increasing, to remove the cell debris.

Next, the THC was observed to increase after feeding of both NPs and LPS injection, but feeding on a diet with Au ions caused the THC to decrease. The number of circulating haemocytes can change dramatically over the course of an infection. In other crustaceans exposed to LPS, haemocyte numbers have been found to increase [81,105] in the hours and days after exposure. However, many studies have also reported significant decreases in the haemocyte count and PO activity after exposure of different crustacean species to environmental pollutants [106]. A low number of circulating haemocytes in crustaceans is strongly correlated with a greater sensitivity to pathogens [107] and, hence, a low THC indicates a higher susceptibility to infectious disease [21]. On the other hand, recruitment of haemocytes, as well as increased phagocytic, phenoloxidase, and antioxidant enzymatic activities, among others, are described as a result of immunostimulantion [63,108]. It remains a challenge for the future research what are the physiological and ecological significances for the organism to sense foreign material in the food.

## Data Availability

Not applicable.

## Appendix A

Figure A1: Physiochemical characterisation of PVP-coated Au NPs and scanning electron microscopy (SEM) of dried leaves covered with PVP–Au NPs. Table A1: Details the number of animals used in the feeding experiment. Table A2: Details the number of animals used in the injection experiment.

**Table A1:** Details the number of animals used in the feeding experiment, the mortality rate, and the stages of the investigation. *, pooled from 4–5 animals; **, pooled from 1–3 animals.

		Biological Repeats (Number of Animals Used)						
Treatment (Feeding)		Number of Animals (Start of 2 Weeks)	Mortality	Removed (Gravid/Molting/Infected)	Feeding Activity	Haemocyte Count	Nitric Oxide *	Phenoloxidase-Like Activity **
**Control**	**Total**	50	4	5	41	28	7 (32)	13 (27)
		30	2	4	24	11		
		20	2	1	17	17	1 (5)	2 (4)
							6 (27)	
								11 (23)
**Cerium NPs**	**Total**	20	3	1	16	16	7 (32)	14 (29)
		20	3	1	16	16	2 (9)	2 (5)
							5 (23)	
								12 (24)
**Gold NPs**	**Total**	30	2	2	26	18	8 (34)	12 (20)
		30	2	2	26	18		
							8 (34)	
								12 (20)
**Gold Ions**	**Total**	30	2	0	28	19	8 (33)	11 (15)
		30	2	0	28	19		
							8 (33)	
								11 (15)

**Table A2:** Details regarding the number of animals used in the injection experiment and the stages of the investigation. *, pooled from 4–5 animals; **, pooled from 1–3 animals.

		Biological Repeats (Number of Animals Used)		
Treatment (Injected)		Haemocyte Count	Nitric Oxide *	Phenoloxidase-Like Activity **
**Control**	**Total**	22	7 (31)	23 (47)
		22		5 (8)
				6 (13)
			7 (31)	5 (13)
				4 (8)
				3 (5)
**LPS**	**Total**	22	7 (35)	23 (61)
		22		
				6 (17)
				5 (14)
			3 (15)	6 (14)
				6 (16)
			4 (20)	
**Cerium NPs**	**Total**	17	8 (38)	12 (26)
		17	2 (9)	9 (19)
			2 (10)	
			4 (19)	
				3 (7)
**Gold NPs**	**Total**	10	5 (24)	12 (22)
		10		5 (9)
			1 (5)	
			4 (19)	7 (13)
